# Supplementary material for: Label-free differential imaging of cellular components in mouse brain tissue by wide-band photoacoustic microscopy
Source: bioRxiv. 2023 Feb 28:2023.02.27.530195. Preprint. [Version 1] doi: 10.1101/2023.02.27.530195 (PMC10002654; doi:10.1101/2023.02.27.530195)
Supplement: Supplement 1 [file NIHPP2023.02.27.530195v1-supplement-1.pdf]

bioRxiv preprint doi: https://doi.org/10.1101/2023.02.27.530195; this version posted February 28, 2023. The copyright holder for this preprint (which was not certified by peer review) is the author/funder, who has granted bioRxiv a license to display the preprint in perpetuity. It is made available under aCC-BY 4.0 International license.

Supplementary Information

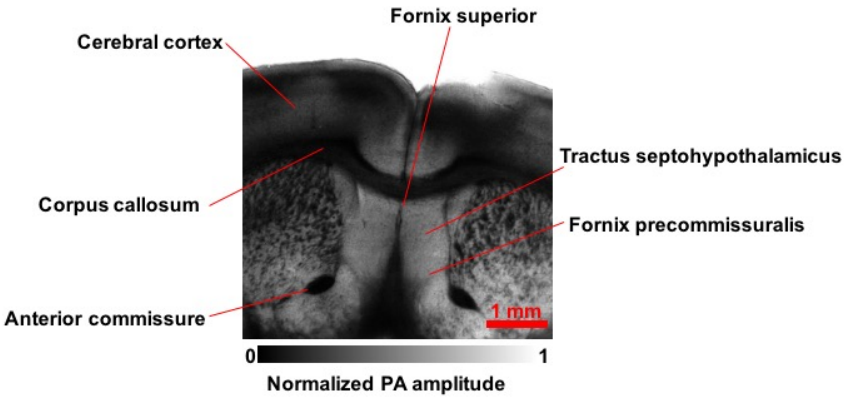

Fig. S1. Label-free wbOR-PAM image of a mouse brain slice acquired at 1720 nm, showing the structure of a mouse brain in coronal view clearly.

bioRxiv preprint doi: https://doi.org/10.1101/2023.02.27.530195; this version posted February 28, 2023. The copyright holder for this preprint (which was not certified by peer review) is the author/funder, who has granted bioRxiv a license to display the preprint in perpetuity. It is made available under aCC-BY 4.0 International license.

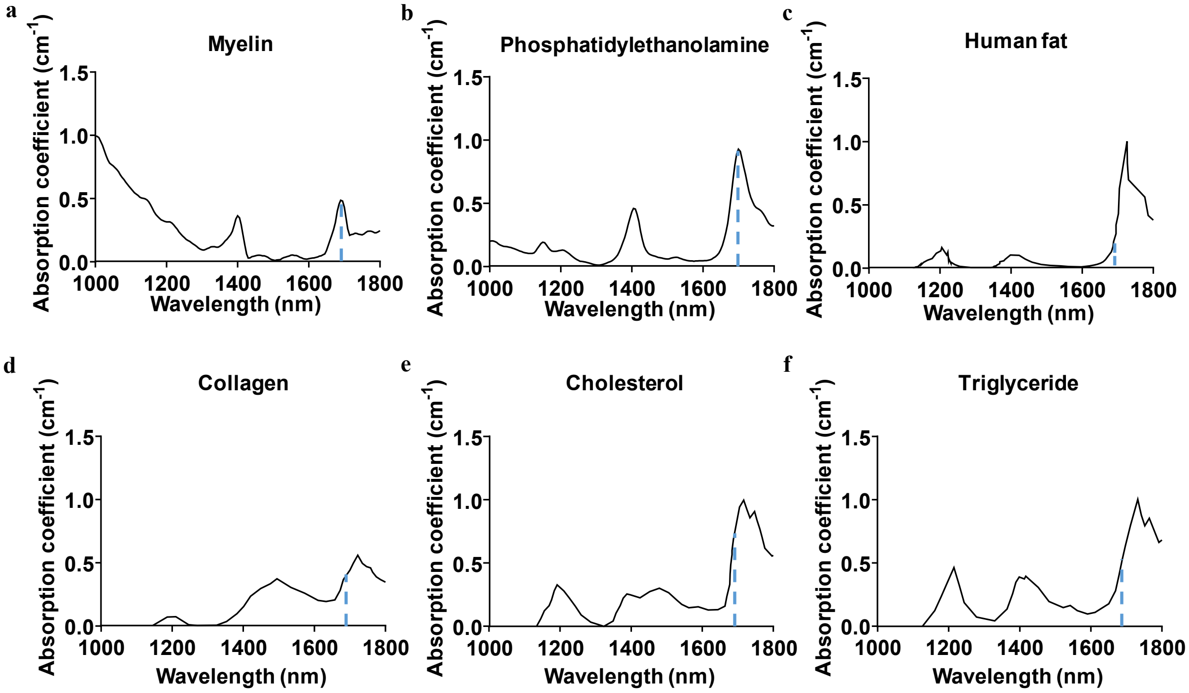

Fig. S2. Absorption spectra of **(a)** myelin, **(b)** phosphatidylethanolamine, **(c)** human fat, **(d)** collagen, **(e)** cholesterol, and **(f)** triglyceride. Blue dashed lines in the spectra denote the absorption coefficients of endogenous contrasts at 1690 nm.

bioRxiv preprint doi: https://doi.org/10.1101/2023.02.27.530195; this version posted February 28, 2023. The copyright holder for this preprint (which was not certified by peer review) is the author/funder, who has granted bioRxiv a license to display the preprint in perpetuity. It is made available under aCC-BY 4.0 International license.

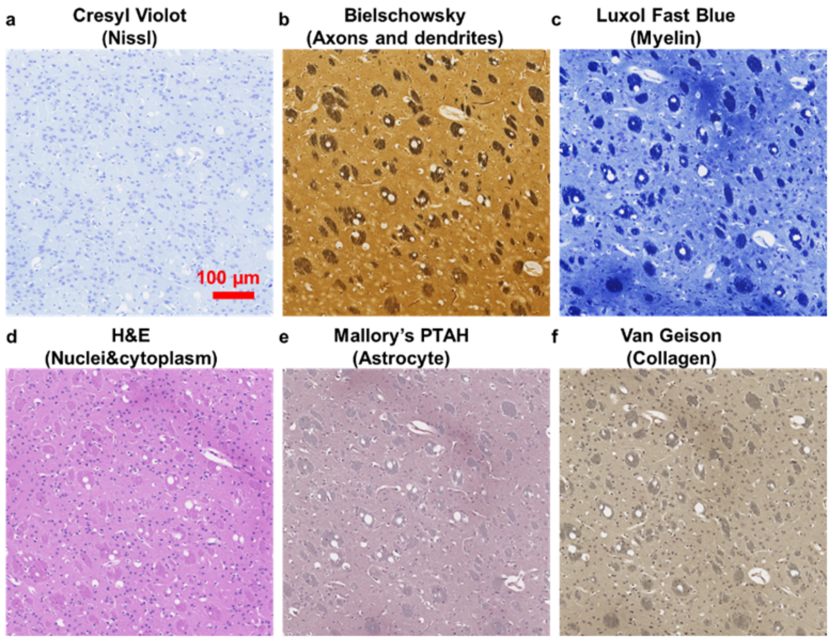

Fig. S3. Histological images of paraffin-embedded brain slices stained with different dyes to highlight **(a)** Nissl, **(b)** Axons and dendrites, **(c)** Myelin, **(d)** Cell nuclei and cytoplasm, **(e)** Astrocyte, and **(f)** Collagen. **(a)–(f)** share the same scale bar (500 μm).

bioRxiv preprint doi: https://doi.org/10.1101/2023.02.27.530195; this version posted February 28, 2023. The copyright holder for this preprint (which was not certified by peer review) is the author/funder, who has granted bioRxiv a license to display the preprint in perpetuity. It is made available under aCC-BY 4.0 International license.

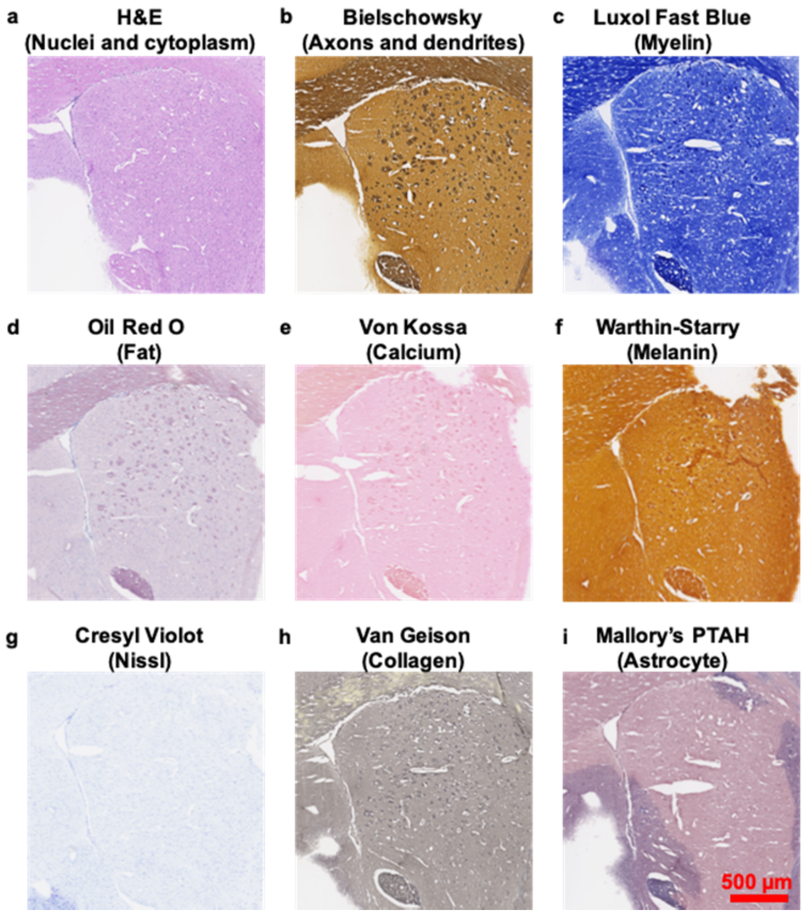

Fig. S4. Histological images of 10-μm thick frozen brain slices stained with different dyes showing **(a)** Nuclei and cytoplasm, **(b)** Axons and dendrites, **(c)** Myelin, **(d)** Fat, **(e)** Calcium, **(f)** Melanin, **(g)** Nissl, **(h)** Calcium, and **(i)** Astrocyte. All the images share the same scale bar marked in **(i)**.

bioRxiv preprint doi: https://doi.org/10.1101/2023.02.27.530195; this version posted February 28, 2023. The copyright holder for this preprint (which was not certified by peer review) is the author/funder, who has granted bioRxiv a license to display the preprint in perpetuity. It is made available under aCC-BY 4.0 International license.

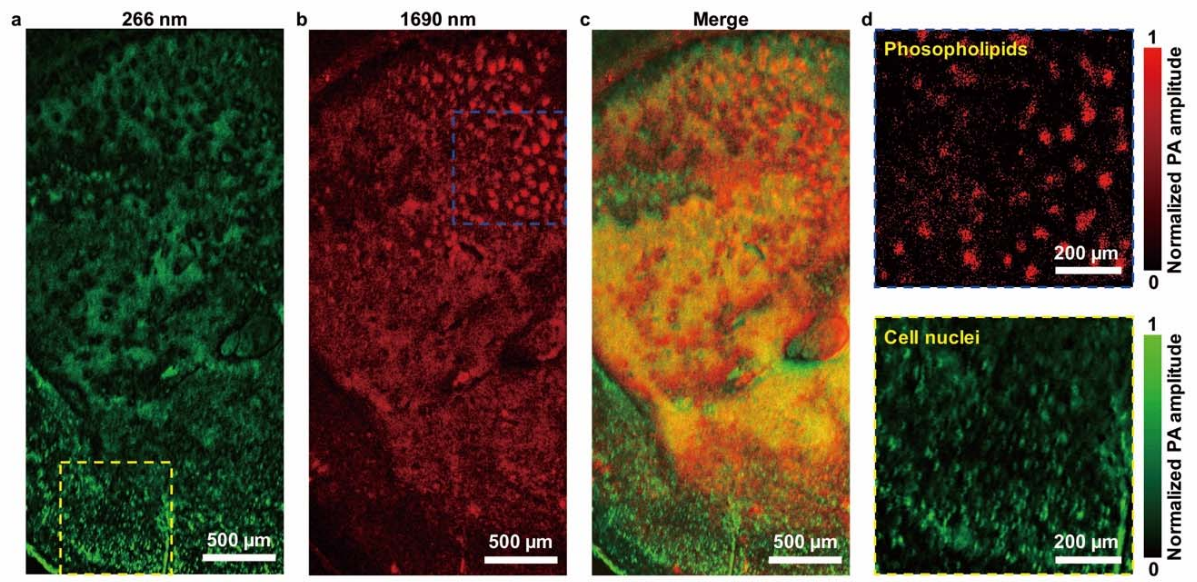

Fig. S5. Cell nuclei and phospholipids in wbOR-PAM images of a 300 μm-thick formalin-fixed mouse brain slice. **(a)** wbOR-PAM image obtained at 266 nm (green) shows cell nuclei. **(b)** wbOR-PAM image obtained at 1690 nm (red) shows the distribution of phospholipids. **(c)** Merged images of **(a)** and **(b)**. **(d)** Close-up images of phospholipids (blue dashed square) in **(b)** and cell nuclei (yellow dashed square) in **(a)**, respectively.

bioRxiv preprint doi: https://doi.org/10.1101/2023.02.27.530195; this version posted February 28, 2023. The copyright holder for this preprint (which was not certified by peer review) is the author/funder, who has granted bioRxiv a license to display the preprint in perpetuity. It is made available under aCC-BY 4.0 International license.

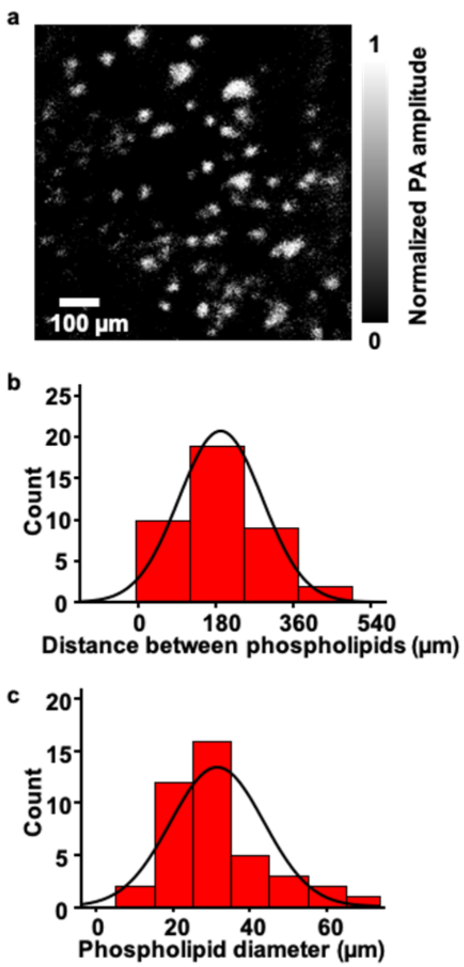

Fig. S6. Distributions of phospholipids in the mouse brain slice. **(a)** wbOR-PAM image of phospholipids, obtained at 1690 nm. **(b)** Histogram of the distance between phospholipids (**n** = 40). The black curve is a Gaussian fit with a mean of 190.7 μm and a standard deviation (SD) of 96.6 μm. **(c)** Histogram of the phospholipids diameter (**n** = 41). The black curve is a Gaussian fit with a mean of 31.6 μm and an SD of 12.2 μm.

bioRxiv preprint doi: https://doi.org/10.1101/2023.02.27.530195; this version posted February 28, 2023. The copyright holder for this preprint (which was not certified by peer review) is the author/funder, who has granted bioRxiv a license to display the preprint in perpetuity. It is made available under aCC-BY 4.0 International license.

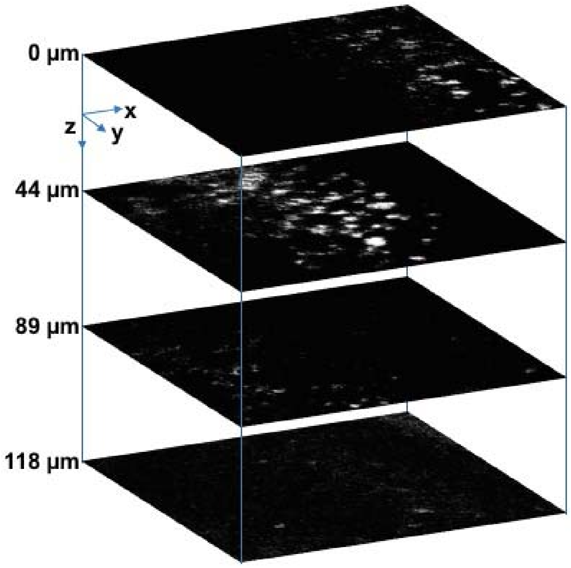

Fig. S7. Label-free wbOR-PAM images of phospholipids in a 300 μm-thick mouse brain slice acquired at 1690 nm in different layers (depths of 0, 44, 89 and 118 μm).
